# Clinical Utility and Usability of the Digital Box and Block Test: Mixed Methods Study

**DOI:** 10.2196/54939

**Published:** 2024-05-23

**Authors:** Eveline Prochaska, Elske Ammenwerth

**Affiliations:** 1 Institute of Medical Informatics, University for Health Sciences, Medical Informatics and Technology, Hall in Tirol, Austria; 2 Institute for Medical Informatics and Biometry, Faculty of Medicine Carl Gustav Carus, Technical University Dresden, Dresden, Germany

**Keywords:** assessment, clinical utility, digital Box and Block Test, dBBT, hand dexterity, dexterity, usability

## Abstract

**Background:**

The Box and Block Test (BBT) is a clinical tool used to measure hand dexterity, which is often used for tracking disease progression or the effectiveness of therapy, particularly benefiting older adults and those with neurological conditions. Digitizing the measurement of hand function may enhance the quality of data collection. We have developed and validated a prototype that digitizes this test, known as the digital BBT (dBBT), which automatically measures time and determines and displays the test result.

**Objective:**

This study aimed to investigate the clinical utility and usability of the newly developed dBBT and to collect suggestions for future improvements.

**Methods:**

A total of 4 occupational therapists participated in our study. To evaluate the clinical utility, we compared the dBBT to the BBT across dimensions such as acceptance, portability, energy and effort, time, and costs. We observed therapists using the dBBT as a dexterity measurement tool and conducted a quantitative usability questionnaire using the System Usability Scale (SUS), along with a focus group. Evaluative, structured, and qualitative content analysis was used for the qualitative data, whereas quantitative analysis was applied to questionnaire data. The qualitative and quantitative data were merged and analyzed using a convergent mixed methods approach.

**Results:**

Overall, the results of the evaluative content analysis suggested that the dBBT had a better clinical utility than the original BBT, with ratings of all collected participant statements for the dBBT being 45% (45/99) equal to, 48% (48/99) better than, and 6% (6/99) lesser than the BBT. Particularly in the subcategories “acceptance,” “time required for evaluation,” and “purchase costs,” the dBBT was rated as being better than the original BBT. The dBBT achieved a mean SUS score of 83 (95% CI 76-96). Additionally, several suggested changes to the system were identified.

**Conclusions:**

The study demonstrated an overall positive evaluation of the clinical utility and usability of the dBBT. Valuable insights were gathered for future system iterations. These pioneering results highlight the potential of digitizing hand dexterity assessments.

## Introduction

Hand function is crucial for performing all activities of daily living [1]. Accidents, injuries, or diseases can lead to limitations in hand function, which need to be assessed in the health care setting. Hand assessment involves a systematic evaluation to quantify and assess the quality of a person’s hand function [2].

**Figure 1.**
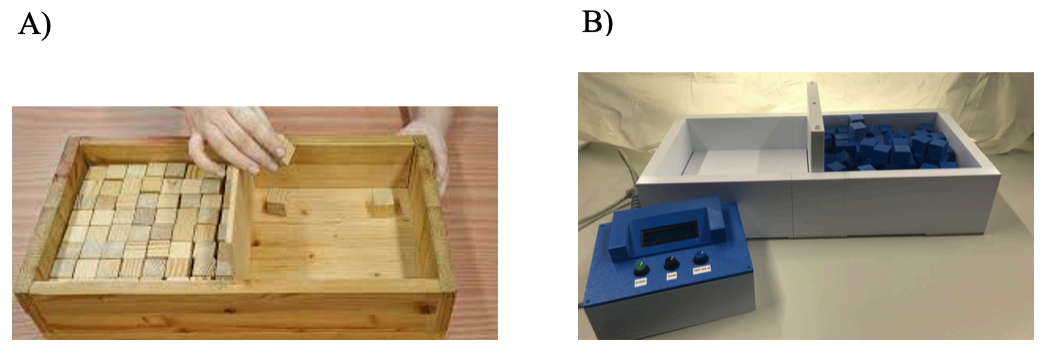
(**A**) The original Box and Block Test and (**B**) the digital Box and Block Test.

The Box and Block Test (BBT) is a widely used assessment for measuring hand dexterity, a crucial aspect of hand function [3]. The original BBT comprises a wooden box with a raised partition at the center (see Figure 1A). The objective is to transfer as many blocks as possible from 1 side of the partition to the other within a 60-second time frame [4]. This assessment, in its unaltered format, has been used for decades, predominantly in clinical settings, to quantify gross manual dexterity [5].

In recent times, several projects have focused on digitizing the BBT to improve the quality of collected data through automated measurement processes [6] or to enable cost-effective home use [7]. Technologies such as depth cameras [8], sensor wristbands [9], and infrared sensors [10] have been used to monitor hand and block movements, providing detailed data on hand dexterity, including kinematic movement profiles [11]. Virtual adaptations of the BBT use leap motion sensors [12], Microsoft Kinect sensors [11,12], or virtual reality headset [3,13-16], eliminating the need for physical BBT materials and offering cost-effective alternatives that are suitable for home use. Additionally, interactive haptic devices provide tactile and force feedback in a virtual environment, aiding in motor function recovery [7].

However, although the advancements offer various advantages, they also present several drawbacks:

- Additional costs: Implementing these advancements can be costly due to the need for extra technologies such as computers, cameras, sensors, and specialized software.
- Additional knowledge: Using technical devices requires extra knowledge, both in operating the systems and managing the increased amount of collected data.
- Increased preparation time: Testers and patients need training before using these methods to ensure the correct handling of the necessary equipment.
- Impact on clinical utility: These new developments sacrifice the simplicity and speed of test performance offered by the original BBT measurement method, potentially affecting their usefulness in clinical settings. However, little attention has been paid to this aspect in previous studies [11].

We have therefore developed the digital BBT (dBBT) with the aim of preserving its clinical utility [17]. This digital adaptation maintains the structural and form aspects of the original BBT while incorporating automated functions for time measurement, cube counting (see Figure 1B), and failure detection. The psychometric properties, including validity, test-retest reliability, and interrater reliability, of the dBBT have been previously examined in a separate study [17]. In addition to validity and reliability, clinical relevance is determined by clinical utility and usability. Hence, this study is focused on assessing the clinical utility and usability of the newly developed dBBT.

When introducing new technology or systems in health care, demonstrating clinical utility is essential. Although widely used, the term “clinical utility” lacks a formal definition [18]. It is used in evaluating clinical effectiveness [19], as well as in economic assessments of costs, benefits, and effectiveness [20]. First et al [21] define it as the degree to which a system aids in various clinical functions. However, this definition overlooks practical, nonclinical concerns.

Simply being valid and reliable does not guarantee clinical usefulness. For instance, therapists may avoid using a test if it is time-consuming or overly complex [22]. Therefore, a comprehensive definition of clinical utility should encompass aspects such as therapist time and ease of use, as outlined by Fawcett [23]. Fawcett’s key dimensions of clinical utility include acceptance, portability, energy and effort, time, and cost.

A usability test is a method of evaluating how user-friendly or intuitive a product is. It involves representative users performing a specific task with the product. Usability tests can be used to identify usability problems, collect data, and determine satisfaction with a product. The System Usability Scale (SUS) is a widely used scale to quantify the usability of many software and hardware products [24]. The SUS was thus selected for this study.

The objective of this study was to evaluate the clinical utility and usability of the dBBT among occupational therapists, who are prospective users. Additionally, the study sought to identify potential areas for future system enhancements.

## Methods

### Participants

The BBT protocol requires a therapist to perform the hand function measurements [5]. Therefore, occupational therapists were selected as the target group for this evaluation. Recruitment took place at the University of Applied Sciences Campus Vienna, with initial outreach conducted by lecturers of the occupational therapy program. Interested individuals were then contacted and provided with study details via email. Inclusion criteria encompassed individuals who (1) were member of the occupational therapy professional group, (2) have practical experience with the original BBT, (3) were at least 18 years old, and (4) have practical experience in the field of occupational therapy and with the BBT.

A total of 4 occupational therapists were recruited. For focus groups, an optimal group size of 4 to 6 participants is recommended [25,26], whereas a minimum of 3 suffices for usability studies [27]. Therefore, a group size of 4 participants was considered adequate for this study.

### Ethical Considerations

The study protocol was conducted in accordance with the Declaration of Helsinki and was approved by the ethics committee (EK Nr 97/2022) of the University of Applied Sciences Campus Vienna. All participants provided written informed consent prior to participation.

### Study Design

**Figure 2.**
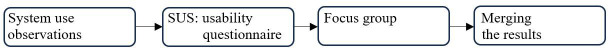
Overview of the mixed methods study design. SUS: System Usability Scale.

The Consolidated Criteria for Reporting Qualitative Research (COREQ) checklist [28] was used for planning, conducting, and reporting this study. This study has been registered on the Open Science Framework [29]. We adopted a mixed methods approach, blending quantitative and qualitative data collection and analysis within a single study [30]. Combining quantitative and qualitative methods typically offers a fuller perspective on the research problem [31]. This study used a convergent mixed methods design, as depicted in Figure 2.

All data collection was overseen by a single researcher (EP), who has been specializing in medical informatics since 2016. The researcher has collaborated with the occupational therapy department on various projects, including the development and ongoing enhancement of the dBBT. Importantly, participants in this study had no prior personal acquaintance with the researcher before recruitment.

The study took place in a laboratory situated at the University of Applied Sciences Campus Vienna, chosen to control for potential confounding variables and enhance result validity. The selection of the study setting was carefully deliberated and considered during implementation.

### Data Collection and Analysis

#### Overview

All data collection and analysis were conducted by a single researcher, with input from a second researcher during the initial coding phase of the data. Data processing and analysis were carried out using MAXQDA 2022 (VERBI GmbH).

For qualitative data, including those from observations and focus groups, an evaluative qualitative content analysis was used [32,33]. This method assessed, classified, and evaluated content, akin to a content-structuring qualitative content analysis. However, in an evaluative content analysis, additional categories are generated to allow researchers to rate the material on the selected dimensions [33-35]. In this research, these assessment dimensions were defined as being less than, equal to, or better than the original BBT measurement instrument. The predefined coding categories in the content analysis process were grounded in 5 key dimensions of clinical utility. Subcategories and guiding questions were subsequently developed for each dimension, serving as the foundation for the observation studies and focus group (see Table 1).

**Table 1.** The dimensions and subcategories of clinical utility (adapted from Fawcett [23]).

Dimensions and subcategories		Description and guiding questions
**Acceptance**		
	Therapists	Is the test administrator (therapist) motivated to work with it? Does he or she enjoy using the measurement instrument?
	Stakeholders	Is the test accepted by clinic management, lay observers, or relatives of clients?
	Clients	Is the test acceptable to clients? Does the test cause stress or test anxiety? Does the client recognize the relevance of the test?
	Professionality	Does the test look professional?
	Face validity	Does the system appear valid? On the surface, does it measure what it is supposed to measure?
**Portability**		
	Clarity of required components	Is it easy to handle in terms of the number of components required?
	Transportability	Can the assessment be transferred from 1 location to another with little effort?
**Energy and effort**		
	Physical exertion	How high is the physical load for the test administrator when performing the test? For example, does the client need to be physically supported?
	Ease of test execution	How easy is it to perform the test? Are there a large number of tasks or extensive material that must be used?
	Ease of learning	How easy is it to learn how to perform the test?
**Time**		
	For learning test execution	How much time is required to learn how to administer and instruct clients on the test?
	For evaluation	How much time is required for the interpretation of the test results?
	For preparation	How much time is required to prepare the test in order to perform the measurement on a client?
	For execution	The most obvious time factor of a measurement procedure [23] How much time is required to perform the test?
**Cost**		
	Ongoing costs	What ongoing costs are incurred for test implementation? (software, test sheets, etc)
	Required training	Are fee-based training courses required for the use of the test?
	Required qualifications	Are there any special qualifications required for test administration or the interpretation of the test results? Must the scoring be performed by specially qualified persons?
	Purchase costs	Which costs are calculated for the acquisition of the test, if necessary for manual and test sheets?

#### System Use Observations

The aim of these observations was to evaluate the clinical utility of the dBBT. Observations in general can provide important real-time data on behavior [36]. The object of observation was the use of the dBBT to measure hand dexterity by a therapist, with another participant as the person to be tested. Each occupational therapist completed the hand dexterity assessment with the dBBT as a test administrator once, whereas another participant took the role of the tested person. The exact procedure was as follows: following the standardized procedure as defined for the original BBT [4], the test administrator read the test instructions to the person to be tested and performed the hand dexterity measurement once on the writing hand of the tested person. The whole exercise session was observed by the researcher, using the previously developed observation guide.

As the observation sequence lasts only a few minutes (including the start-up of the dBBT by the therapist, instruction of the participant by the therapist, practice run following the test protocol, and the actual dexterity test), there was limited time for extensive note-taking. Therefore, the researcher opted for a quantitative assessment of the observations. The following aspects of the five dimensions of clinical utility were assessed, which were directly observable and comparable to the original BBT using a three-part scale of less than, equal to, or better than: (1) time for preparation by the therapist, (2) time for patient instruction by the therapist, and (3) time for the person to be tested to understand the test task. Further assessment points covered possible application problems: (4) problems during preparation (which ones, severity, and consequences), (5) problems during implementation (which ones, severity, and consequences), and (6) open questions from the therapist (see ).

Each session was video recorded using a Dell Latitude 5480 Laptop, and data were collected using the aforementioned observation protocol.

The data were transcribed verbatim, anonymized, and coded using predefined categories (see Table 1), and additional categories were developed based on the data (for potential improvements to the dBBT). Subsequently, the data were analyzed using verbal-interpretative methods based on the categories, and key statements were presented accordingly. All these steps were carried out by the same person, the researcher (EP).

#### Usability Questionnaire

The usability of dBBT was then assessed quantitatively with the SUS (see Multimedia Appendix 2). The participants (N=4) completed the SUS directly after using the dBBT.

The SUS is one of the most frequently used questionnaires for evaluating the usability of eHealth applications [37]. Even with a very small sample, the SUS provides valid results about whether an application has problems in the area of usability [38].

The process for computing a SUS score was following: (1) subtract 1 from the Likert ratings for odd-numbered items, (2) subtract the Likert ratings from 5 for even-numbered items (each item score will range from 0 to 4), and (3) sum the numbers and multiply the total by 2.5 [24]. This resulted in SUS scores ranging from 0 to 100. The following SUS score ratings were used in this study: scores ≥52 represented “OK” usability, ≥73 represented “good” usability, and ≥85 represented “excellent” usability [39].

The mean SUS score from 3500 surveys within 273 studies was 72 [39]. It is recommended to the report mean, SD, median, CI, and *P* value (Shapiro-Wilk) in addition to the SUS score [24].

#### Focus Group Interview

Following the completion of the usability questionnaire, a focus group session was conducted with all 4 occupational therapists. The aim was to evaluate the clinical utility of the dBBT compared to the original version and to gather suggestions for potential system improvements.

The focus group followed a predefined guideline (see Multimedia Appendix 3), developed in accordance with qualitative research standards [40] and reviewed by a second researcher. This guideline was structured around the dimensions of clinical utility (see Table 1), with the assessment criteria for the evaluative content analysis (less than, equal to, or better than the BBT) also included.

An audio recording was made during the focus group session. This audio file, an observation protocol created by the researcher following the focus group, and notes from the guideline were used for data analysis. For analysis, an evaluative qualitative content approach was chosen [33]. An initial coding frame was derived from the focus group guideline and refined as more data were analyzed. This involved identifying patterns, assigning codes, and establishing themes and subthemes from the coded data [41]. Ultimately, the data were interpreted verbally according to categories and presented alongside relevant statements.

#### Merging Qualitative and Quantitative Data

We used a convergent mixed methods design, integrating the findings from both qualitative and quantitative data [42]. Following separate collection and analysis of quantitative and qualitative information, the 2 data sets were combined.

The purpose of merging the results was twofold: (1) to enhance the validation of clinical utility and usability and (2) to identify potential optimization strategies.

The following data were included in the merging process:

- Qualitative results from the focus group and observations
- Quantitative results from the observations (observation protocols, see Multimedia Appendix 1) and usability questionnaires (see Multimedia Appendix 2)

Subsequently, recommendations for future improvements were extracted from the compiled data and presented.

## Results

### Overview

The studies were conducted in April 2023. The focus group lasted approximately 1.5 hours, the observational studies lasted a total of 10 minutes for the dexterity measurements, and the SUS required 5 minutes per person. All 4 occupational therapists involved in the study were between 21 and 30 years old, and all participants were female.

This section is divided into three subsections: (1) results for clinical utility, (2) results for usability, and (3) recommendations for potential future changes for the dBBT. In the subsection on clinical utility, statements regarding each of the 5 aspects of clinical utility are highlighted, representing the obtained results. Quotes are labeled with their corresponding line numbers in the audio transcript. The assessment of usability follows immediately afterward. Finally, the section concludes with recommendations, presenting a concise list of potential optimization measures identified for the dBBT based on the validation results.

### Clinical Utility

#### Acceptance

The acceptance of the newly developed prototype dBBT differed from the original BBT in several ways. First, simplicity was enhanced by eliminating the need for manual counting (with the original BBT, the therapist has to count the transported blocks by hand to obtain a final test result; on average, 80-100 blocks have to be counted by hand, which makes the evaluation more time-consuming than the BBT itself) and by automating time measurement instead of using a stopwatch: “for me the automatic time measurement easier than dealing with a stopwatch - because I just never use a stopwatch otherwise” (p.25).

Second, the trustworthiness of the results provided by the dBBT was emphasized, ensuring that the results are credible to clients: “and above all, that the result [note: for clients] is credible - if a ‘device’ measure that” (p.125).

Another important finding was the clinical and professional appearance of the dBBT, which was documented in several statements, such as “[the dBBT] transports a higher level of professionalism to the external environment” (p.128).

The evaluative analysis showed that the dBBT achieved higher acceptance compared to the BBT. As shown in Table 2, a total of 89% (33/37) of statements by the occupational therapists showed a higher acceptance of the dBBT than the original BBT. Particularly, all occupational therapists judged the “professionalism” (defined as whether the test looks professional [13]) of the dBBT as higher than that of the original BBT.

**Table 2.** The clinical utility of the digital Box and Block Test (dBBT) as expressed by occupational therapists. “Less than,” “equal to,” and “better than” indicate their evaluated statements for the dBBT (ie, the dBBT performs less than, equal to, or better than the original Box and Block Test).

Dimensions and subcategories		Statements on the dBBT with rating^a^			
		Total, n^b^	Less than, n (%)^c^	Equal to, n (%)^c^	Better than, n (%)^c^
**Acceptance**					
	By administrator	10	1 (10)	0 (0)	9 (90)
	By stakeholder	6	0 (0)	1 (17)	5 (83)
	By patients	8	1 (12)	1 (12)	6 (75)
	Professionality	8	0 (0)	0 (0)	8 (100)
	Face validity	5	0 (0)	0 (0)	5 (100)
	Total	37	2 (5)	2 (5)	33 (90)
**Portability**					
	Clarity of components	1	0 (0)	1 (100)	0 (0)
	Transportability	8	3 (38)	5 (62)	0 (0)
	Total	9	3 (33)	6 (67)	0 (0)
**Energy and effort**					
	Physical exertion	2	0 (0)	2 (100)	0 (0)
	Ease of execution	2	0 (0)	2 (100)	0 (0)
	Ease of learning	3	1 (33)	2 (67)	0 (0)
	Total	7	1 (14)	6 (86)	0 (0)
**Time**					
	Learning test execution	3	0 (0)	3 (100)	0 (0)
	For evaluation	4	0 (0)	0 (0)	4 (100)
	For preparation	6	0 (0)	6 (100)	0 (0)
	For execution	11	0 (0)	11 (100)	0 (0)
	Total	24	0 (0)	20 (83)	4 (17)
**Cost**					
	Ongoing costs	8	0 (0)	2 (25)	6 (75)
	Required training	8	0 (0)	7 (88)	1 (12)
	Required qualifications	2	0 (0)	2 (100)	0 (0)
	Purchase costs	4	0 (0)	0 (0)	4 (100)
	Total	22	0 (0)	11 (50)	11 (50)
Total		99	6 (6)	45 (45)	48 (48)

a Due to rounding, percentages may not sum to 100%.

b Overall number of statements for the respective item, both in the observation analysis or focus group.

c Percentages are calculated with the values in the “Total, n” column as the denominators.

#### Portability

The clarity of the components was rated as equal to the BBT, but the transportability of the dBBT was rated as lesser than that of the BBT. This is because the original BBT can be folded to half its size, whereas the dBBT does not offer this feature: “possibly it is bulkier the dBBT” (p.92).

Regarding the clarity of required components (“Is it easy to handle in terms of the number of components required?”), the dBBT was rated as equal to the BBT: “so there is no difference to the BBT - except that you don’t have to assemble the dBBT - then the dBBT is even rather clearer.” (p.116).

The dBBT is slightly heavier than the BBT. However, the therapists came to the conclusion that the higher weight of the dBBT is irrelevant because “normally the BBT will not be transported either - it will be in the therapy room” (p.112).

According to the evaluative analysis results for portability, 67% (6/9) of statements reported that the portability of the dBBT was equal to that of the BBT. The remaining statements (3/9, 33%) suggested that the dBBT had less transportability than the BBT (Table 2).

#### Energy and Effort

In most of the statements in the energy and effort category and its subcategories (physical exertion, ease of test execution, and ease of learning), no difference was found between the dBBT and BBT: “so certainly none of the three aspects [note: of energy and effort] shows somehow more effort or energy than with the BBT” (p.108) and “I would see it the same way” (p.109).

The energy required to perform the measurement with the dBBT and to learn how to perform dexterity measurement with the dBBT was also rated as equal to that for the BBT: “the physical effort for the test administrator is the same as for a measurement with the BBT” (p.105) and “the effort required for implementation is the same, learning is just as easy as with the BBT” (p.110).

In the evaluative analysis results for energy and effort in Table 2, a total of 86% (6/7) of the statements reported equal energy and effort in using the dBBT in comparison to the BBT.

#### Time

In the subcategory of time for evaluation, the dBBT was rated as better than the BBT by all participants. The therapists appreciated the simplification resulting from the omission of counting the blocks, especially when evaluating multiple patients consecutively: “then I would also prefer the digital BBT - because it would be tedious to count and check it all the time” (p.52) and “slightly less time for the evaluation of the test with the dBBT than with the BBT” (p.96).

In the subcategories for learning test execution, preparation, and implementation, the dBBT was rated as equal to the BBT (Table 2): “I only see a little less time for the evaluation - everything else is the same” (p.86) and “the time to learn how to perform the test cannot be different” (p.98).

The preparation and execution of the hand function measurement with the dBBT were evaluated as equal to the BBT: “you have to plug in the dBBT and try it out, I guess – but at the BBT I have to check whether the stopwatch is working” (p. 89-90) and “The preparation is also no different than with the BBT - put it there…” (p.88).

In the evaluative analysis results for time in Table 2, a total of 83% (20/24) of statements in the time category rated the dBBT as equal to the BBT, whereas 17% (4/24) rated the dBBT as better than the BBT.

#### Cost

The BBT is available for purchase at prices ranging from US $240 to US $450. The new dBBT was estimated to cost a fraction of this amount. The manufacturing costs are estimated to be less than US $65. This information was given to the test participants before the discussion of costs.

The ongoing costs for dBBT were estimated to be relatively lower (if one classified the power consumption as negligible): “less are the running costs with the dBBT - because I don’t need a battery for the stopwatch” (p.78).

Regarding necessary training, all therapists were receptive to the fact that the technical device requires minimal additional effort due to its straightforward operation. However, it was noted that an introduction to the functions of the dBBT was required initially: “I think it balances out - since you don’t have to count with the dBBT. that’s not necessary. but the three buttons and plugging the device in [to the power supply] are the ‘more’ - but once you know it, you can do it anyway” (p.17) and “you have to be told at least once what, for example, the black button is for and so on” (p.19).

At the same time, however, familiarity with using a stopwatch, which was required for the original BBT, had decreased: *“*the stopwatch I need to use in the original, I also need to practice” (p.22). Therefore, the expense of required training was rated as equal to the BBT.

The required qualification for executing a dexterity measurement with the dBBT was rated as equal to the BBT: “the qualification for the admin is the same, as the standardized test protocol is just as possible with the dBBT as with the BBT” (p.5).

In total, 50% (11/22) of statements in the cost category rated the dBBT as equal to the the original BBT and 50% (11/22) rated it as better than the BBT. The purchase cost of the dBBT was considered better than the BBT, whereas in all other subcategories, the dBBT was considered equal to the BBT.

#### Summary

The evaluative qualitative content analysis used selected dimensions (less than, equal to, and better than) to assess the clinical utility of the dBBT compared to the original BBT measurement instrument. In summary, 45% (45/99) of all statements reported an equivalent assessment of the dBBT. Furthermore, 48% (48/99) of all statements rated the clinical utility of the dBBT as better than that of the dBBT, whereas only 6% (6/99) of the statements rated the dBBT as less than the BBT (refer to Table 2).

Therefore, in this study, the dBBT consistently appeared to have at least comparable, and often superior, results in terms of clinical utility compared to the BBT.

### Usability

Usability was evaluated using the standardized SUS. The SUS was administered immediately following the observation study. Consequently, participants engaged in a standardized hand dexterity assessment (in a laboratory setting) before evaluating the dBBT using the SUS. Table 3 presents the survey results obtained after the observations.

**Table 3.** System Usability Scale (SUS) score for the digital Box and Block Test from 4 therapists.

Metric	SUS score
Mean (SD)	83 (10)
Median (range)	87.5 (72.5-95)
95% CI	76-96

### Recommendations

Several themes regarding potential future changes for the dBBT emerged from observations and the focus group discussion. A total of 3 points for potential improvements had been identified, supported by collected data and defined recommendations.

#### Theme 1: Shape of the Blocks

The most commonly suggested improvement for the system pertained to the shape of the blocks. Participants expressed that making the edges less sharp would enhance the ease of handling the blocks: “The cubes are more difficult to grip [the edges are sharper than on the BBT]…“ (p.10) and “...Edges are sharper or very sharp in the dBBT, which means that they are arranged more closely in the box and it is harder to grip them” (p.13).

- Recommendation 1: Adaption of edge shapes of the dBBT, by making the edges rounder

#### Theme 2: Additional Acoustic Signal for Test Ending

The second point focused on signaling the end of the test period. Currently, the dBBT uses 2 LEDs, placed on the partition, that change from green to red when the 60-second test period concludes. However, the participants preferred an audible signal, as it would provide a clearer notification for both the person being tested and the therapist: “…the stopwatch beeps so nicely [note: on the original BBT] - then the patient knows that the measurement period is over” (p.80) and “…that would also be good if this prototype can do that…” (p.81).

Additionally, it was observed in 3 (75%) out of 4 instances, the visual signal for the test ending was not perceived by either the test administrator or the person being tested.

- Recommendation 2: The implementation of an acoustic signal that marks the end of the test period

#### Theme 3: Continuous Display During Test Run

The third point became evident from observations alone. During the test, 2 (50%) out of the 4 occupational therapists were distracted by the display, which constantly showed the elapsed time and the number of blocks currently being counted. The standardized test procedure requires occupational therapists to ensure that the person being tested (1) crosses the partition with their fingers and (2) transports only one block at a time. However, constantly checking the changing display diverted the therapists’ attention from this task. None of the participants in the focus group mentioned this issue. It is possible that this observed behavior is a result of using a new device, and whether this problem persists with continued use of the dBBT cannot be conclusively answered by this study alone.

- Recommendation 3: Deactivate the continuous display during the test procedure; activate the display only at the start and end of the test

No other subthemes regarding future changes were found.

## Discussion

This study is the first thorough assessment of the clinical effectiveness and user-friendliness of the dBBT, revealing user opinions and possible advantages of such systems. Apart from insights into its clinical utility and usability, the findings present valuable perspectives from end users that can shape the future development of digital assessments.

### Clinical Utility

Clinical utility plays a pivotal role in selecting and using a measurement technique. The original BBT is well regarded for its characteristics: quick administration, simplicity, clinic-friendliness, and portability [4,5,43,44]. However, using a wooden measuring tool with a stopwatch is outdated now. Switching to digital methods for measuring hand dexterity can enhance data collection quality [6] and increase acceptance among both patients and therapists. However, these advantages matter only in the health care sector if digitalization does not make measuring hand dexterity more complicated.

To evaluate the clinical utility of the dBBT, we divided it into different aspects based on existing literature. These aspects encompassed acceptance, portability, energy and effort, time, and cost, totaling 17 subcategories [23]. We assessed these aspects in comparison to the original BBT, using a 3-point scale (less than, equal to, and better than).

The dBBT surpassed the original BBT in terms of acceptance (across all 5 subcategories) and in the subcategories of evaluation time and purchase costs. Compared to the BBT, the dBBT garnered higher acceptance from users and patients. The improved rating in evaluation time is attributed to the fact that the test administrator no longer needs to manually tally the approximately 80-100 transported blocks after completing the test. The results are automatically recorded and displayed, saving the time required for measurement. The assessment of the notably lower purchase cost of the dBBT is grounded in a comparison between the costly BBT, as outlined in the *Results* section, and the estimated manufacturing expenses of the dBBT.

In summary, the comparison of the clinical utility of the BBT and dBBT revealed superior results for the dBBT in terms of acceptance, time, and costs. The results were comparable in the dimensions of energy and effort, whereas the BBT demonstrated better results in transportability.

### Usability

The usability of the dBBT was evaluated using the SUS, a standardized and validated tool even with small random samples [38]. All participants provided data immediately after using the dBBT, which was then quantitatively analyzed. The mean SUS score for the dBBT was 83 (SD 10). This result exceeded the mean SUS score of 72 from 237 studies for hardware [39]. Since a SUS score of 85 or higher is considered excellent, the outcomes are highly favorable [39].

### Future Work

The systematic evaluation of the dBBT has generated valuable insights for future system iterations. Subsequent efforts will be directed toward incorporating these enhancements into the system. Moreover, future endeavors will concentrate on gauging users’ perceptions of the system within authentic clinical settings and through prolonged use over time. This approach will enable the objective assessment of the system’s influence on users in clinical environments and facilitate a comparison with the perceived impact identified in this study.

### Comparison With Prior Work

In the early stages of digital innovation, understanding usability from an end user’s perspective is critical. Active and early involvement of users in the design process helps identify unforeseen user experience issues, enhancing user engagement, a crucial factor in overall user acceptance [38]. Assessing clinical utility is essential for a comprehensive evaluation of assessments [23].

Several publications discuss advancements in various versions of the BBT, integrating additional technologies such as sensors, cameras, or virtual reality [3,8,10,11,13,15,16]. However, there remains a lack of validation regarding the clinical utility of digitized measurement instruments [11].

One study examined the perceived user-friendliness and acceptance of a virtual BBT using a satisfaction questionnaire, yielding highly positive results for the examined development [13]. Another study, using the Intrinsic Motivation Inventory, reported greater motivation with the virtual BBT compared to the traditional BBT [7]. However, Cho et al [11] noted reduced accessibility with a further virtual iteration of the BBT due to the additional technical equipment required. To our knowledge, no studies have explored the clinical advantages of newly developed digital versions of the BBT using the dimensions proposed by Fawcett [23].

Everard et al [3,14] reported comparable usability results, with SUS scores of 78 and 83 among healthy participants using a virtual BBT. At the time of this study, no additional results on the usability of digitized BBTs were available.

### Clinical Implications

During development, researchers should not only consider the functionality of a new system but also its practicality. Without the cooperation and acceptance of users, the functionality of a new system may prove ineffective [6].

Overall, the data suggested that the dBBT prototype maintained the fundamental advantage of the BBT (simplicity and quick execution) despite its digitization.

The measurement of hand dexterity with the dBBT adheres to the standardized test protocol of the BBT. Given that the BBT and its testing procedure are widely used and well-known among clinicians, the adoption of the dBBT as a measurement tool is straightforward. There is no need to develop new descriptions for test procedures and patient instructions, as these are readily available for the BBT and are equally applicable to the dBBT.

With its automated measurement of time and results, the dBBT holds significant potential for resource savings in research. The automated measurement can minimize variability among different testers, thereby enhancing data quality. Moreover, the high acceptance among all participants can yield additional benefits for clinical practice.

The advantages of the dBBT can enhance the assessment of hand dexterity in health care. The dBBT has the potential to become a complementary tool for clinical practice.

### Limitations

Several contextual factors should be considered when interpreting our findings. All results in this study reflected participants’ first experiences with the system. Although this approach is suitable for identifying perceived clinical utility and usability issues, it is possible that perceptions may evolve over time. Further studies are warranted to explore the long-term clinical utility and usability of the dBBT.

Additionally, this study was conducted in a controlled laboratory environment. Evaluating the system in real clinical settings may uncover additional usability and functionality issues, as well as opportunities for further improvements.

Due to the early stage of development, the involvement of patients was rejected for ethical reasons. This combined with the small sample size and homogeneity of participants may limit the generalizability of results, particularly when considering diverse demographics or populations with varying levels of interest in technology.

Data collection, transcription, and analysis were performed by a single researcher, with the first coding of the data supported by feedback from a second researcher. Although there was high consistency in merging the quantitative and qualitative results, it is important to acknowledge the potential influence of a single researcher.

Furthermore, this paper primarily focused on assessing the clinical utility and usability of the dBBT. Extensive details on the psychometric properties of the dBBT are available in a recent publication by the authors [17].

### Conclusions

In conclusion, this study serves as a pioneering exploration into the clinical utility and usability of the dBBT, offering valuable insights into user perspectives and the potential advantages of digital assessment systems.

This research sheds light on the promising prospects of the dBBT in terms of clinical utility and usability, acting as a bridge between traditional assessments and digital innovations. As we further refine and broaden our understanding of this digital tool, the dBBT holds significant potential for enhancing hand dexterity assessments in clinical practice, benefiting both patients and health care providers.

## Supplementary material

10.2196/54939 Multimedia Appendix 1 Observation guideline.

10.2196/54939 Multimedia Appendix 2 System Usability Scale.

10.2196/54939 Multimedia Appendix 3 Focus group guideline.

## Abbreviations

BBT: Box and Block Test
COREQ: Consolidated Criteria for Reporting Qualitative Research
dBBT: digital Box and Block Test
SUS: System Usability Scale
