# Supplementary material for: Clinical Utility and Usability of the Digital Box and Block Test: Mixed Methods Study
Source: JMIR Rehabil Assist Technol. 2024 May 23;11:e54939. doi: 10.2196/54939 (PMC11137429; doi:10.2196/54939)
Supplement: Multimedia Appendix 1 [file rehab-v11-e54939-s001.docx]

**Observation Guideline**

Task: Performance of a hand dexterity measurement with dBBT - according to standardized specifications.

Observation start:

1. Therapist places subject in front of the measurement device (dBBT)

2. instruction is read out by the administrator

3. test of writing hand (15 seconds)

4. test of writing hand (60 sec.)

End of observation: after completion of the test

Estimated observation time (per setting): 3 to 4 minutes.

Dimensions of observation:

1. Time for preparation (Time): time needed by therapist for startup (comparable to original BBT, longer, shorter)

| less | equal | better |
| --- | --- | --- |

1. Time for learning test execution (Time): therapist time required for patient instruction (comparable to original BBT, longer, shorter)

| less | equal | better |
| --- | --- | --- |

1. Ease of learning (Energy and Effort): time needed by the client to understand the task (comparable to original BBT, longer, shorter)

| less | equal | better |
| --- | --- | --- |

1. problems with start-up (type, severity, consequences)
2. problems with performing (type, severity, consequences)
3. open questions of the occupational therapists
