# Supplementary figures and images for: Clinical Utility and Usability of the Digital Box and Block Test: Mixed Methods Study
Source: JMIR Rehabil Assist Technol. 2024 May 23;11:e54939. doi: 10.2196/54939 (PMC11137429; doi:10.2196/54939)

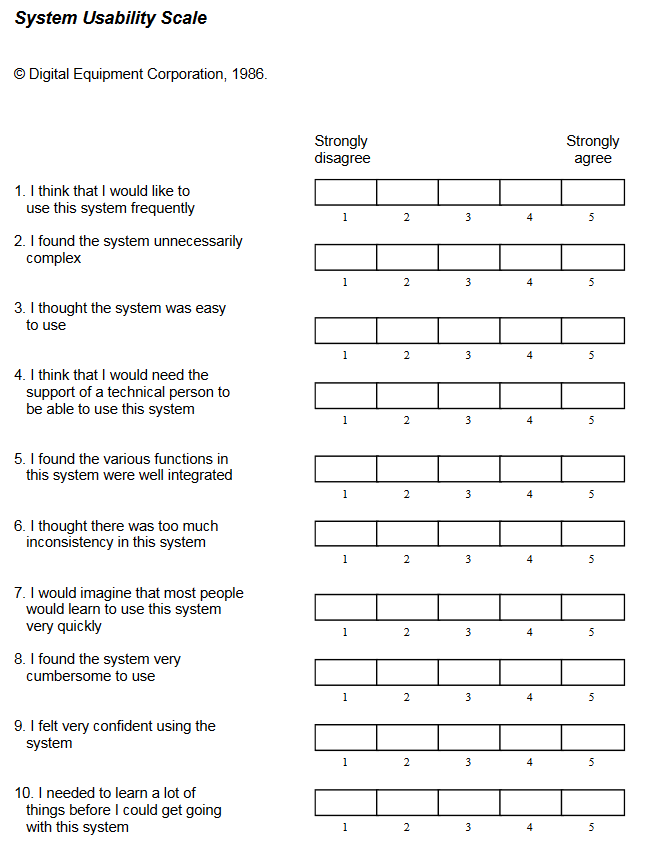

Supplement: Multimedia Appendix 2 [file rehab-v11-e54939-s002.docx]
