# Supplementary material for: Clinical Utility and Usability of the Digital Box and Block Test: Mixed Methods Study
Source: JMIR Rehabil Assist Technol. 2024 May 23;11:e54939. doi: 10.2196/54939 (PMC11137429; doi:10.2196/54939)
Supplement: Multimedia Appendix 3 [file rehab-v11-e54939-s003.docx]

Guideline focus group

| Categories | Description / guiding questions | less | equal | better |
| --- | --- | --- | --- | --- |
|  |  | in comparison with the original BBT | | |
| Acceptance |  |  |  |  |
| Therapists | Is the test administrator (therapist) motivated to work with it?  Does he enjoy using the measurement instrument? |  |  |  |
| Stakeholders | Is the test accepted by clinic management, lay observers or relatives of clients? |  |  |  |
| Clients | Is the test acceptable to clients?  Does the test cause stress or test anxiety?  Does the client recognize the relevance of the test? |  |  |  |
| Professionality | Does the test look professional? |  |  |  |
| Face validity | Does the system appear valid? On the surface, does it measure what it is supposed to measure? |  |  |  |
| Portability | |  |  |  |
| Clarity of required components | Is it easy to handle in terms of the number of components required? |  |  |  |
| Transportability | Can the assessment be transferred from one location to another with little effort? |  |  |  |
| Energy and Effort | |  |  |  |
| Physical exertion | How high is the physical load for the test admin when performing the test?  For example, does the client need to be physically supported? |  |  |  |
| Ease of test execution | How easy is it to perform the test?  Are there a large number of tasks or extensive material that must be used? |  |  |  |
| Ease of learning | How easy is it to learn how to perform the test? |  |  |  |
| Time | |  |  |  |
| for learning test execution | How much time is required to learn how to administer and instruct clients on tests? |  |  |  |
| for evaluation | How much time is required for interpretation of test result? |  |  |  |
| for preparation | How much time is required to prepare the test in order to perform the measurement on a client? |  |  |  |
| for execution | The most obvious time factor of a measurement procedure[13]. How much time is required to perform? |  |  |  |
| Cost | |  |  |  |
| Ongoing costs | What ongoing costs are incurred for test implementation? (software, test sheets,...) |  |  |  |
| Required training | Are fee-based training courses required for the use of the test? |  |  |  |
| Required qualifications | Are there any special qualifications required for test administration or for interpretation of the test results?  Must the scoring be performed by specially qualified persons? |  |  |  |
| Purchase costs | Which costs are calculated for the acquisition of the test, if necessary for manual and test sheets? |  |  |  |
